# Diffuse cystic adenomyosis simulating invasive uterine neoplasm on imaging: A postmenopausal diagnostic perplexity!

**DOI:** 10.18632/oncoscience.615

**Published:** 2025-02-10

**Authors:** Anusha Devalla, Mishu Mangla, Krishna Ramavath, Shailaja Prabhala, Naina Kumar, Aparna Jarathi

**Affiliations:** ^1^Department of Obstetrics and Gynecology, All India Institute of Medical Sciences, Bibinagar, Hyderabad 508126, India; ^2^Department of General Surgery, All India Institute of Medical Sciences, Bibinagar, Hyderabad 508126, India; ^3^Department of Pathology, All India Institute of Medical Sciences, Bibinagar, Hyderabad 508126, India

**Keywords:** postmenopausal, hyperplasia, aged, 80 and over, endometrial neoplasms, adenomyosis

## Abstract

Postmenopausal bleeding (PMB) with a diffusely enlarged uterus necessitates Magnetic Resonance Imaging (MRI) to reach an accurate diagnosis. Adenomyosis, especially extensive glandular variant, is an extremely rare cause reported in a postmenopausal woman. We present a challenging case of an 81-year-old woman with PMB where preoperative MRI suggested possible invasive endometrial neoplasm. However, final histopathological evidence of the hysterectomy specimen suggested Adenomyosis with extensive glandular proliferation. The patient was a multiparous lady with controlled diabetes and hypertension (controlled on medications) and a Body Mass Index of 36 kg/m^2^. Bimanual examination suggested a diffusely enlarged uterus corresponding to 8-10 weeks gestation. Transvaginal ultrasound (TVUS) and Contrast Enhanced (CE) MRI were performed that reported multiple cystic areas with myometrial thinning at the fundal region- suspected infiltrating endometrial neoplasm. A hysteroscopic guided endometrial biopsy was suggestive of endometrial hyperplasia. In view of concerning MRI findings, a total abdominal hysterectomy and bilateral Salpingo-oophorectomy was performed. Histopathological examination revealed Adenomyosis with extensive glandular proliferation co-existing with endometrial hyperplasia and no atypia. This case highlights an important variant of Adenomyosis that potentially mimics an invasive uterine neoplasm. There is a dearth of uniform reporting standards for Adenomyosis and rarity of this condition in postmenopausal woman posing a significant preoperative diagnostic challenge.

## INTRODUCTION

Postmenopausal bleeding with an enlarged uterus needs thorough clinical, radiological and histopathological correlation [1]. Various causes ranging from atrophic vaginitis and endometritis to endometrial cancer have been well established. Adenomyosis as a cause of postmenopausal bleeding is uncommon, hysterectomy specimens are 0.49% [2–4]. Age more than 40 years, previous uterine surgeries, multiparity are some of the risk factors of adenomyosis. Despite advances in imaging modalities, diagnosis of Adenomyosis still needs histological correlation. Magnetic Resonance Imaging (MRI) offers sensitivity rates of up to 88 % and specificity rates of up to 93 % in diagnosis Adenomyosis. The characteristic appearance of a diffusely adenomyotic uterus shows thickened myometrium trabeculations around ectopic endometrial glands. Extensive glandular proliferation is an extremely rare variant of Adenomyosis which has been reported in only one case of a premenopausal woman. To the best of our knowledge, the authors report a first ever case of extensive glandular proliferation in Adenomyosis that mimicked malignancy on MRI in an 81-year-old postmenopausal woman.

## CASE REPORT

An 81-year-old multiparous woman of Asian ethnicity presented to gynecological outpatient department with recurrent postmenopausal bleeding episodes for the past 3 months. She had no associated white discharge per vaginum, pain in abdomen and associated altered bladder or bowel symptoms, or loss of appetite. She was a known diabetic and hypertensive, controlled on medications. She attained menopause 20 years back. Her past menstrual cycles were irregular and heavier, associated with clots and dysmenorrhea. She took symptomatic treatment from a local practitioner without undergoing a formal evaluation. There was no family history of any medical comorbidities or malignancies. On examination, patient had BMI of 36 kg/m^2^ (Class II obesity), and abdominal examination was unremarkable. Speculum examination showed a healthy cervix and a small polyp 2 × 2 cm arising in the right lateral wall of vagina. On pelvic examination, the uterus was 8–10 weeks size, mobile and bilateral fornices were free and non-tender, cervix firm and regular. She had no palpable lymph nodes. Cervical smear for cytology showed no malignancy. Her routine preoperative investigations suggested hemoglobin, renal and liver function tests, sugars and thyroid functions were within normal limits.

A Two-dimensional Transvaginal Ultrasonography (2D-TVS) of pelvis was done that suggested uterus 82 mm × 57 mm × 51 mm and thickened endometrium 29 mm and multiple cystic areas with myometrial thinning at the fundal region-a diagnosis of suspected endometrial neoplasm with myometrial invasion was made. Bilateral ovaries and adnexa were unremarkable.

**Figure 1:**
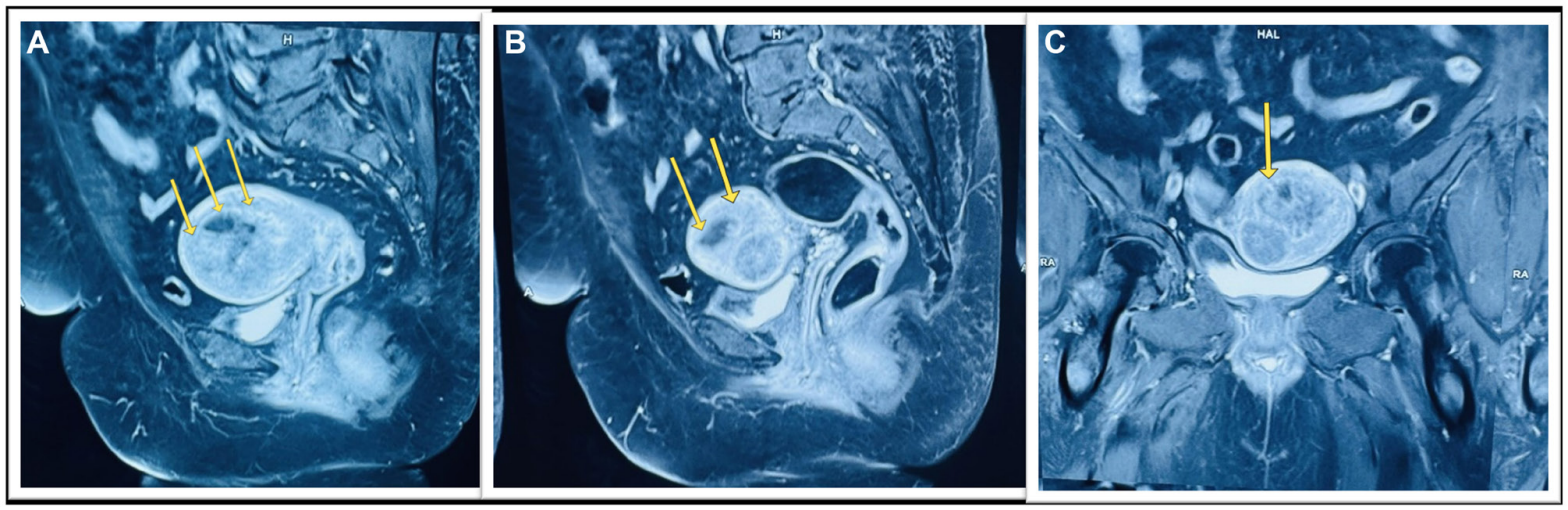
(**A**) Shows the T2 sagittal 3T Contrast enhanced Magnetic Resonance Imaging (CEMRI) pelvis showing cystic endometrial hyperplasia/endometrial neoplasm and myometrial thinning with loss of junctional zone (shown by yellow arrows); (**B**) Diffusion Weighted Imaging (DWI) and Apparent Diffusion Coefficient of 0.85 (shown by yellow arrows); (**C**) Shows transverse plane CEMRI pelvis showing myometrial thinning.

To further confirm the diagnosis, a Contrast-enhanced pelvic Magnetic Resonance Imaging (CEMRI) showed the presence of T2 hyperintense foci in the junctional zone and myometrium of fundus and upper uterine body anteriorly with evidence of effusion restriction with Apparent Diffusion Coefficient of 0.87 × 10^−3^ mm^2^/s on Diffusion-Weighted Imaging (DWI) (Figure 1). Endometrium evaluation through hysteroscopic guided biopsy revealed a diffuse fluffy hyperplastic polypoidal endometrium and mild increased vascularity with histological changes of non-atypical endometrial hyperplasia. Microscopic examination of the excised vaginal polyp revealed a fibroepithelial polyp.

**Figure 2:**
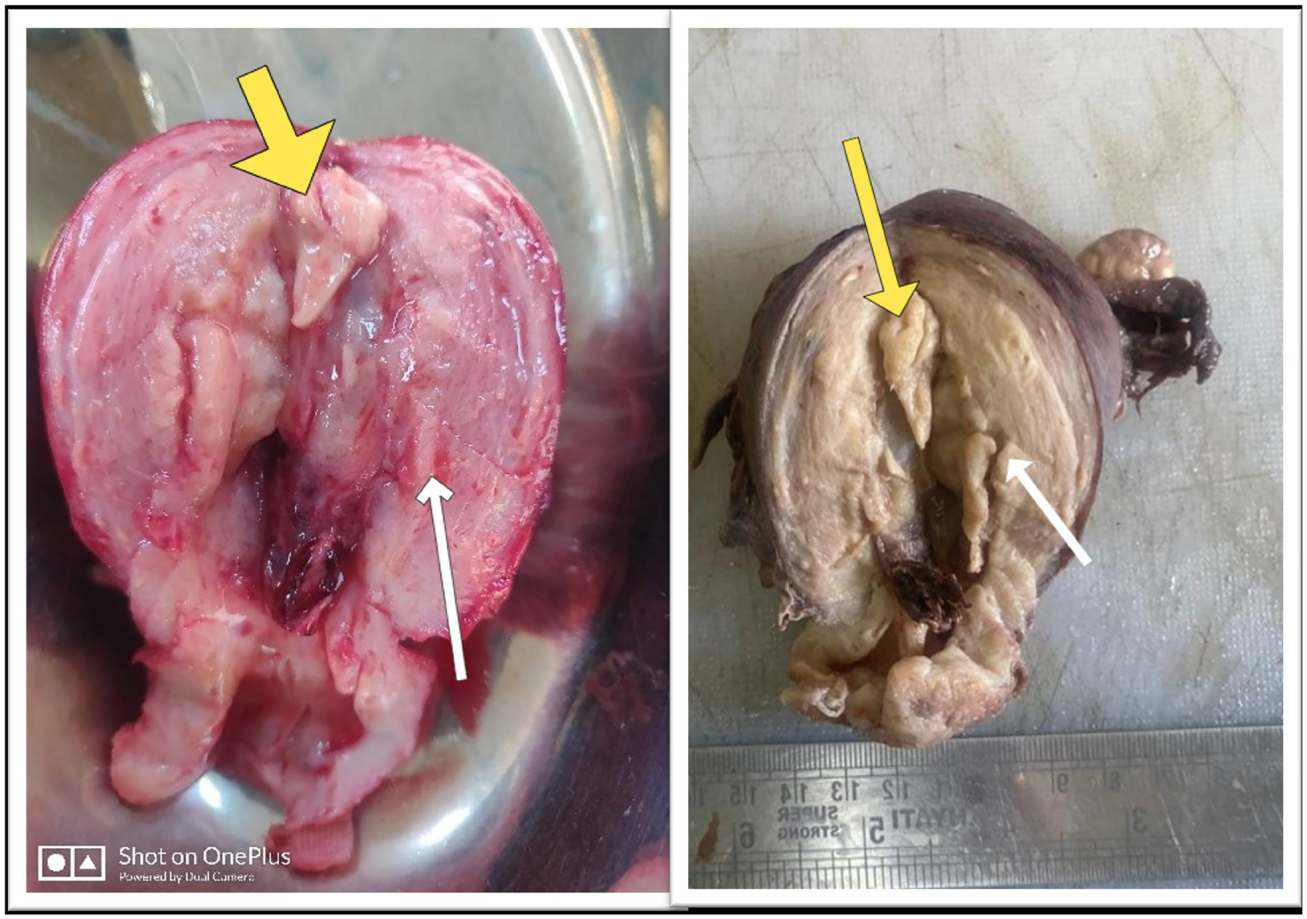
(**A**) Cut-open hysterectomy specimen (uterus with cervix) showing fundal polyp (marked with yellow arrow) with overlying thin myometrium; Diffuse hyperplastic endometrium (marked with white arrow); (**B**) Formalin fixed hysterectomy specimen (weight 110 gms).

In view of the concerning findings on MRI and the characteristic clinical profile, a total abdominal hysterectomy with bilateral salpingo-oophorectomy was performed (Figure 2). There were no palpable para-aortic and pelvic lymph nodes. Intraoperatively cytological examination of the peritoneal washings was negative for malignant cells.

**Figure 3:**
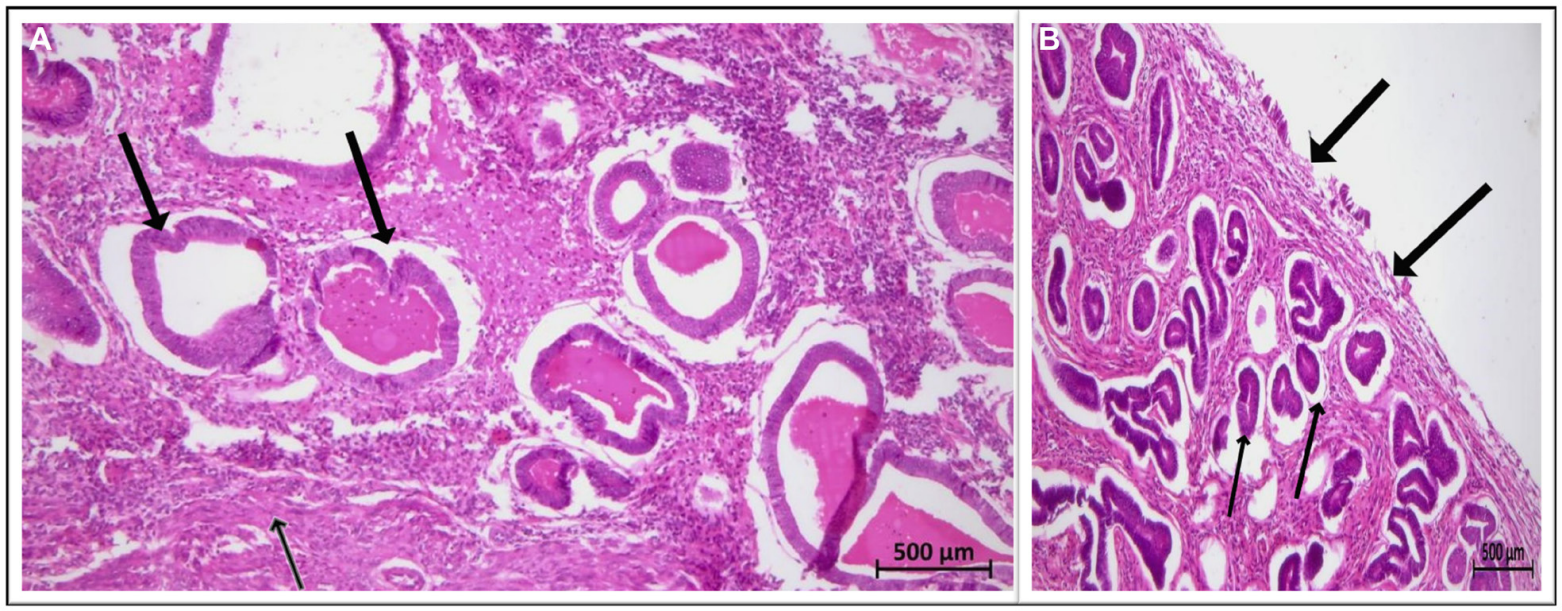
(**A**) Adenomyosis with glands (Thick arrow) in the myometrium (thin arrow). (5x, H and E stain); (**B**) Endometrial hyperplasia with surface lining on the right (Thick arrows), the gland to stroma ratio is increased and glands show proliferative features with round to tubular glands having stratification of cells (Thin arrow) (50X, H and E stain).

Gross specimen showed the uterine size to be around 9 × 7 × 6 cm^3^ and the cut section revealed multiple polypoidal growths with thinning noted near fundal region, raising the suspicion further. Contrary to the imaging findings, the histopathological examination revealed extensive glandular proliferation variant of adenomyosis co-existing with non-atypical endometrial hyperplasia (Figure 3). Post-operative recovery was uneventful with no abnormality noted on subsequent follow up at one month.

## DISCUSSION

We report a challenging case of extensive glandular proliferation variant of Adenomyosis in an 81-year-old postmenopausal woman. To the best of our knowledge, this case highlights the presence of this rare variant of extensive glandular proliferation in adenomyosis in a geriatric 81-year-old woman. A recent mention by N Kumar et al. in 2024 witnessed a similar presentation in a 64-year-old woman with adenomyosis detected on histopathology with preoperative MRI showing diffusion restriction and ADC of 0.6 × 10^−3^ mm^2^/s [5].

Adenomyosis may coexist with a number of pathologies, namely, leiomyomas, endometrial hyperplasia, endometrial polyps [6]. Our case also highlights the presence of vaginal fibroepithelial polyp with concurrent endometrial hyperplasia to be hyperestrogenic milieu, rarely observed in a postmenopausal woman. Similarly, K Funaki et al. in 2011 reported a case of 40-year-old with a florid presentation on gross findings mimicking invasive uterine malignancy that was subsequently diagnosed as multicystic Adenomyosis [7].

The varied presentation of Adenomyosis has posed a huge diagnostic dilemma for the clinicians and the radiologists especially with atypical appearances on MRI showing myometrial invasion and diffusion restriction highly indicative of uterine malignancy. On T2-weighted magnetic resonance (MR) images, typical adenomyosis appears as an ill-demarcated low-signal-intensity lesion with uterine enlargement [8]. This was unlike in our case where the T2 DWI images showed hyperintensity of the myometrium and loss of junctional zone. On T2-weighted images, tumour signal intensity ranged from hyperintensity to slight hypointensity compared with the moderate signal intensity of the myometrium. On dynamic and postcontrast Ti-weighted images, viable tumours had intermediate signal intensity compared with the well-enhanced myometrium and with the hypointense endometrial cavity. If endometrial cancer cannot be ruled out with definitive histopathological diagnosis in the preoperative period, a frozen section becomes mandatory during surgical intervention in an otherwise young patient [9]. Considering the patient’s preoperative age and clinical spectrum, it was imperative to perform a complete hysterectomy with bilateral salpingo-oophorectomy.

Susceptibility weighted MR and MR spectrometry offers as a better diagnostic modality than the conventional T2 MRI study as the former can differentiate adenomyosis from tumour-like outgrowths. Recently, Morphological Uterus Sonographic Assessment (MUSA) criteria has been introduced to classify the condition on ultrasonography but is not yet widely employed [10].

## CONCLUSIONS

Adenomyosis should be considered as one of the differential diagnosis where the imaging shows blurring of junctional zone with myometrial invasion in the background of endometrial hyperplasia as co-existence with adenomyosis is not uncommon. Better reporting protocols and advanced equipment are necessary which reiterates the need to report such cases.

## References

[1] Miyazawa K. Clinical significance of an enlarged uterus in patients with postmenopausal bleeding. Obstet Gynecol. 1983; 61:148–52. 6823356

[2] Benagiano G, Brosens I. History of adenomyosis. Best Pract Res Clin Obstet Gynaecol. 2006; 20:449–63. 10.1016/j.bpobgyn.2006.01.007. 16515887

[3] Krentel H, De Wilde RL. Prevalence of adenomyosis in women undergoing hysterectomy for abnormal uterine bleeding, pelvic pain or uterine prolapse - A retrospective cohort study. Ann Med Surg (Lond). 2022; 78:103809. 10.1016/j.amsu.2022.103809. 35734686 PMC9206934

[4] Pushpalatha K, Kalra R, Singh B, Devalla A. Rare complication of adenomyosis: acute purulent peritonitis and septicaemia in a young nulligravida. BMJ Case Rep. 2021; 14:e238374. 10.1136/bcr-2020-238374. 34844956 PMC8634357

[5] Kumar N, Sharma A, Mangla M, Srirambhatla A. Adenomyosis or endometrial carcinoma? Radiological pitfalls in postmenopausal diagnosis: a case report. Egyptian Journal of Radiology and Nuclear Medicine. 2024; 55:229. 10.1186/s43055-024-01399-5.

[6] Singh G, Cue L, Puckett Y. Endometrial Hyperplasia. Treasure Island (FL): StatPearls Publishing; 2024. https://www.ncbi.nlm.nih.gov/books/NBK560693/. 32809528

[7] Funaki K, Fukunishi H, Maeda T, Ohbayashi C, Yamaguchi S. Adenomyosis with extensive glandular proliferation simulating infiltrating malignancy on magnetic resonance imaging. Jpn J Radiol. 2011; 29:272–75. 10.1007/s11604-010-0538-6. 21607841

[8] Takeuchi M, Matsuzaki K. Adenomyosis: usual and unusual imaging manifestations, pitfalls, and problem-solving MR imaging techniques. Radiographics. 2011; 31:99–115. 10.1148/rg.311105110. 21257936

[9] Erguvan R, Meydanli MM, Alkan A, Edali MN, Gokce H, Kafkasli A. Abscess in adenomyosis mimicking a malignancy in a 54-year-old woman. Infect Dis Obstet Gynecol. 2003; 11:59–64. 10.1155/S1064744903000085. 12839634 PMC1852262

[10] Van den Bosch T, Dueholm M, Leone FP, Valentin L, Rasmussen CK, Votino A, Van Schoubroeck D, Landolfo C, Installé AJ, Guerriero S, Exacoustos C, Gordts S, Benacerraf B, et al. Terms, definitions and measurements to describe sonographic features of myometrium and uterine masses: a consensus opinion from the Morphological Uterus Sonographic Assessment (MUSA) group. Ultrasound Obstet Gynecol. 2015; 46:284–98. 10.1002/uog.14806. 25652685

